# Single and Combined Serum Proteins Expressed in TB Infection are Candidates for Point-of-care Diagnostic Testing of Active TB Patients in Lambaréné, Gabon

**DOI:** 10.1093/ofid/ofae399

**Published:** 2024-07-13

**Authors:** Paulin N Essone, Fabrice Lotola-Mougeni, Bayode R Adegbite, Kossiwa Kokou, E Otogo N'Nang, Eddy Mabicka, Ayodele Alabi, Joel F Djoba Siawaya, Peter G Kremsner, Martin P Grobusch, Selidji T Agnandji

**Affiliations:** Centre de Recherches Médicales de Lambaréné, Biomedicine and Social Sciences Research Group, Department of Biologicals and Therapeutics, Lambaréné, Gabon; Unité de Recherche et de Diagnostics Spécialisés, Laboratoire National de Santé Publique/Centre Hospitalier Universitaire Mère Enfant Fondation Jeanne EBORI, Libreville, Gabon; Centre de Recherches Médicales de Lambaréné, Biomedicine and Social Sciences Research Group, Department of Biologicals and Therapeutics, Lambaréné, Gabon; Centre de Recherches Médicales de Lambaréné, Biomedicine and Social Sciences Research Group, Department of Biologicals and Therapeutics, Lambaréné, Gabon; Institut für Tropenmedizin, Universität Tübingen and German Center for Infection Research Tübingen, Tübingen, Germany; Department of Infectious Diseases, Center of Tropical Medicine and Travel Medicine, Amsterdam University Medical Centers, location University of Amsterdam, Amsterdam Infection & Immunity, Amsterdam Public Health, University of Amsterdam, Amsterdam, The Netherlands; Centre de Recherches Médicales de Lambaréné, Biomedicine and Social Sciences Research Group, Department of Biologicals and Therapeutics, Lambaréné, Gabon; Centre de Recherches Médicales de Lambaréné, Biomedicine and Social Sciences Research Group, Department of Biologicals and Therapeutics, Lambaréné, Gabon; Centre de Recherches Médicales de Lambaréné, Biomedicine and Social Sciences Research Group, Department of Biologicals and Therapeutics, Lambaréné, Gabon; Centre de Recherches Médicales de Lambaréné, Biomedicine and Social Sciences Research Group, Department of Biologicals and Therapeutics, Lambaréné, Gabon; Unité de Recherche et de Diagnostics Spécialisés, Laboratoire National de Santé Publique/Centre Hospitalier Universitaire Mère Enfant Fondation Jeanne EBORI, Libreville, Gabon; Centre de Recherches Médicales de Lambaréné, Biomedicine and Social Sciences Research Group, Department of Biologicals and Therapeutics, Lambaréné, Gabon; Institut für Tropenmedizin, Universität Tübingen and German Center for Infection Research Tübingen, Tübingen, Germany; Centre de Recherches Médicales de Lambaréné, Biomedicine and Social Sciences Research Group, Department of Biologicals and Therapeutics, Lambaréné, Gabon; Institut für Tropenmedizin, Universität Tübingen and German Center for Infection Research Tübingen, Tübingen, Germany; Department of Infectious Diseases, Center of Tropical Medicine and Travel Medicine, Amsterdam University Medical Centers, location University of Amsterdam, Amsterdam Infection & Immunity, Amsterdam Public Health, University of Amsterdam, Amsterdam, The Netherlands; Centre de Recherches Médicales de Lambaréné, Biomedicine and Social Sciences Research Group, Department of Biologicals and Therapeutics, Lambaréné, Gabon; Institut für Tropenmedizin, Universität Tübingen and German Center for Infection Research Tübingen, Tübingen, Germany; Institute of Medical Microbiology, University Hospital Münster, Münster, Germany

**Keywords:** Biomarkers for tuberculosis, Sulfotransferase 4A1, Wiskott-Aldridge syndrome protein family member 3

## Abstract

**Background and Objectives:**

Point-of-care testing using nonsputum samples like serum or plasma proteins can improve tuberculosis (TB) patients access to a definitive diagnosis, especially in resource-constrained and remote areas. Recently, approximately 400 proteins were identified as playing a role in the pathogenesis of TB, offering a translational clinical research repository for TB. In a previous manuscript, we proved the potential use of these proteins for point-of-care testing for active TB diagnosis. The present work aims to confirm the performance of single and combination proteins to select the best candidate biomarkers for further development as a diagnostic testing tool for active TB.

**Methods:**

Seventy-four participants were assessed on the diagnostic performance of 17 single proteins and combinations of 2 to 4 proteins to diagnose active TB. The selection criteria included differential expression of the proteins between active TB and community-acquired pneumonia (CAP) and a performance rate ≥70% for active TB.

**Results:**

SULT4A1, WASPF3, SPTLC1, FAM107B, SORCS2, and CYTOb561 were differentially expressed in TB compared to CAP patients. Two single proteins, SULT4A1 and WASPF3, performed ≥70% to discriminate active TB from CAP patients. The diagnostic performance of 3 protein-based combinations of active TB was 81% after leave-one-out cross-validation.

**Conclusion:**

Single proteins and 3 protein-based combinations are candidate biomarkers for diagnosing active TB disease. A large and prospective study will confirm their performance as complementary diagnostic tools to rapid diagnostic methods for detecting active TB.

The current armamentarium to confirm active tuberculosis (TB) includes smear microscopy, culture, rapid molecular tests, and lipoarabinomannan antigen detection [1]. Sputum-based smear microscopy, which has a sensitivity of approximately 50%–70% to detect *Mycobacterium tuberculosis* (Mtb), was used for two-thirds of people diagnosed in 2021, even though the World Health Organization (WHO) recommended rapid molecular tests for initial TB diagnosis in 2013 [2]. Yet, high cost and sourcing problems for equipment, materials, and qualified staff; a need for electricity, temperature controls, manual pipetting of samples across instruments; and contamination and difficulties in the maintenance of instruments are, among others, severe hurdles to the scale-up of rapid molecular methods [2]. Consequently, less than 50% of TB cases were diagnosed using WHO-recommended rapid diagnostic tests, and approximately 20%–40% of TB active cases were left undiagnosed in 2023 [1–3].

Tuberculosis infection is a lasting immune response to *Mtb* antigens associated with the absence of clinical signs and symptoms of TB disease, with normal images on chest X-ray and negative bacteriological tests when performed [4]. Worldwide, approximately 5%–10% of TB-infected people develop TB disease in their lifetime [2]. The WHO approves the following host markers to detect TB infection: tuberculin skin test based on intradermal injection with killed Mtb, the interferon-gamma release assays following stimulation of blood samples with the early secretory antigenic 6 kDa protein, or the culture filtrate protein 10, as well as, more recently, intradermal skin tests targeting the early secretory antigenic 6 kDa and culture filtrate protein 10 antigens [4]. No host biomarker is recommended for the diagnosis of active TB. Serum or plasma proteins are deemed highly appropriate for point-of-care testing, such as lateral flow tests based on capillary blood collection [5]. This is important because there is evidence that plasma proteins are involved in TB infections as well as in TB disease through various mechanisms, including inflammation, tissue repair, matrix-remodelling, elevated interferon responses, activation of the complement pathway [6], antimicrobial defense, and disease severity [7]. The value of different plasma/serum protein-based signatures to diagnose active TB has been demonstrated in several studies [7–10]. In a recent breakthrough discovery study of unbiased screening of more than 3000 proteins, 361 proteins were differentially expressed between progressors and nonprogressors. Two biomarkers of either 3 or 5 proteins predicted the occurrence of active TB [11]. We have previously reported the proof of concept that single proteins and a combination of 3 proteins reached good accuracy to distinguish TB from other community-acquired pneumonia (CAP) [12]. In the present manuscript, we report the process of selecting single proteins and protein-based combinations as candidate “biomarkers” to diagnose active TB [2, 4, 13].

## MATERIALS AND METHODS

### Study Design

#### A Cross-sectional Study to Assess the Concentration and Performance of Proteins as a Diagnostic Testing Tool

The cross-sectional study took place in the study area of the Centre de Recherches Médicales de Lambaréné (CERMEL), which includes Lambaréné town and surrounding villages situated within a radius of approximately 70 km in both southerly and northerly directions. Two district hospitals, the Albert Schweizer Hospital and the Georges Rawiri Hospital, of approximately 30 beds each, provide care to patients according to national guidelines. CERMEL hosts the national TB reference laboratory. People who attended outpatient and inpatient clinics in 1 of the 2 hospitals and presented with symptoms and signs of TB were referred to the CERMEL TB laboratory. For this study, all patients referred to the laboratory between January 2019 and December 2020 who tested positive and were otherwise eligible were invited to participate after signing the informed consent form. We enrolled 37 patients who were positive for sputum smear microscopy and Mtb culture and/or GeneXpert MTB/RIF (Cepheid, Sunnyvale, CA, US, Xpert MTB/RIF), as well as 37 who were TB-free but were diagnosed by the treating physician with CAP. The preceding manuscript has described the study population. The blood sample processing and the enzyme-linked immunosorbent assay were used to assess the performance of 4 preselected serum proteins [12]. For this set of proteins, we applied the random forest classification combined with the leave-one-out cross-validation (LOOCV) procedure to estimate the performance of the proteins to classify the cases of TB versus CAP patients and a more reliable and unbiased inference of the results from small sample size to the population.

#### Translation of Discovery Results: Criteria to Identify Proteins to be Assessed in Diagnosing Active TB

We applied the following criteria to preselect proteins for evaluation of their performance as active TB diagnostic testing tools: differential expression in TB patients versus TB infection with fold change from infection to active disease state ≥1.4 and a low false-discovery rate as ≤0.05.

#### Criteria of Selection of Candidates' Biomarker of Active TB

A single protein differentially expressed between active TB and CAP and with a performance rate ≥70% after the LOOCV was selected as a candidate biomarker of active TB. Combinations of at least 2 proteins comprising at least 1 protein differentially expressed in patients with active TB and a performance ≥80% were also selected as biomarker candidates.

## RESULTS

### A Cross-sectional Study in TB and CAP Patients

TB and CAP patients showed different demographic characteristics, including median age, sex ratio, and body mass index at diagnosis (Table 1).

**Table 1.** Demographic Characteristics of Study Participants

	All	No TB	TB	*P* Value
	N = 74	N = 37	N = 37	
Age median [range]	38 [28–53]	45 [33–59]	33 [26–44]	.010
BMI, median [IQR]	19.53 [17.76–21.96]	20.3 [18.30–22.82]	18.52 [16.89, 20.44]	.004
Sex	…	…	…	.035
Female (%)	33 (45)	21 (57)	12 (32)	…
Male (%)	41 (55)	16 (43)	25 (68)	…

Chi-square test was used to compare proportions; Kruskal-Wallis test compared the medians.

Abbreviations: BMI, body mass index; IQR, interquartile range.

### Serum Proteins Tested as Biomarker Candidates for Active TB Diagnosis

From the discovery study results [11], we preselected 31 serum proteins differentially expressed in TB patients compared to TB-infected individuals whose infection did not progress to active TB disease. No robust measurement tool was available for the 10 preselected proteins; hence, 21 proteins underwent the selection process. Four were evaluated and reported in a previously published manuscript [12]. The serum concentrations of 4 proteins were undetectable. The performance of active TB diagnostic testing of 13 additional proteins is reported in the present manuscript (Supplementary Table 1).

### The Performance of Single Proteins as a Diagnostic Testing Tool of Active TB

**Figure 1.**
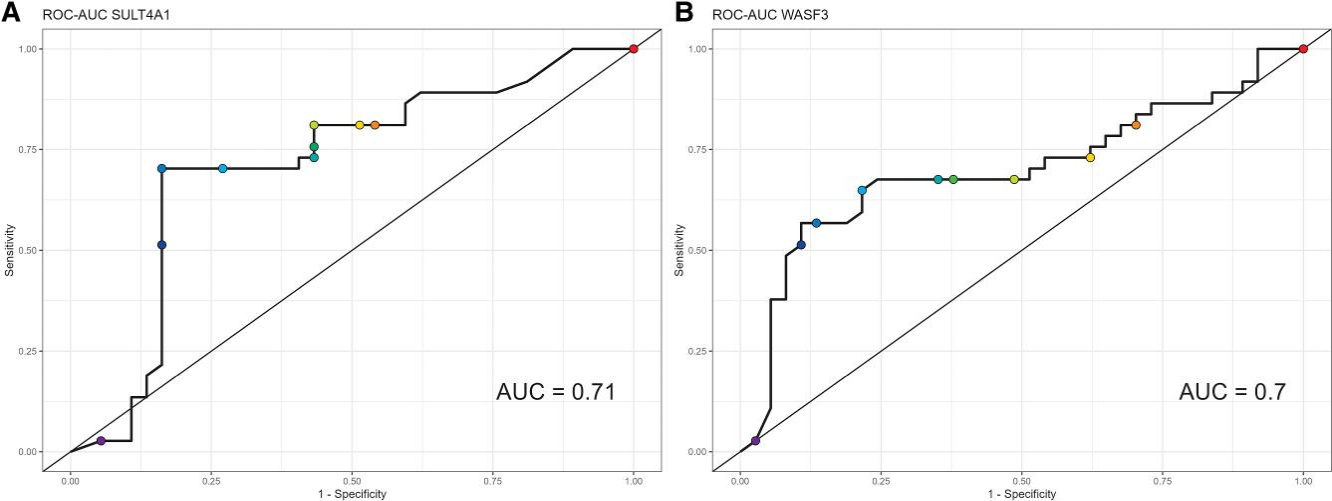
(*A*) (ROC-AUC SULT4A1) and (*B*) (ROC-AUC WASPF3) shows curves of the 2 single proteins SULT4A1and WASPF3 that discriminate TB and CAP patients. Dot plots represent the thresholds used to calculate the true-positive rate (sensitivity) and false-positive rate (1—specificity).

The concentration of sulfotransferase 4A1 (SULT4A1), Wiskott-Aldridge syndrome protein family member 3 (WASF3), family with sequence similarity 107B (FAM107B), serine palmitoyl transferase, long chain base subunit 1 (SPLTC1), and cytochrome B 561 (CYTO b 561) were significantly higher in TB patients compared to CAP patients (Table 2). The sortilin-related VPS10 domain-containing receptor 2 (SORCS2) concentration was lower in TB patients compared to CAP patients (Table 2). When the data were further analyzed using the receiver operating characteristic curve after the LOOCV procedure, 2 proteins, SULT4A1 and WASPF3, reached a performance ≥70% to distinguish TB from CAP patients (Figure 1).

**Table 2.** Eligibility Criteria for Selection of Proteins: Differential Expression and Performance Metrics of Sele

Markers	TB patients N = 37	CAP patients, N = 37	*P*-value^a^	After LOOCV		
				AUC	Sensitivity 95% CI	Specificity 95% CI
SULT4A1	9 (6, 33)^b^	5 (4, 9)^b^	<.001	70 [57–82]	70 (55–84)	57 (41–72)
WASF3	470 (365, 1000)	350 (284, 454)	.003	71 [60–82]	67 (52–81)	64 (49–79)
FAM107B	239 (160, 575)	141 (126, 234)	.002	63 [50–76]	49 (33–64)	62 (47–77)
SPTLC1	8 (4, 24)	2 (2, 8)	.001	64 [51–76]	57 (41–72)	57 (41–72)
CYTOb561	27 (20, 44)	21 (0, 38)	.049	51 [38–64]	49 (33–64)	64 (49–79)
SORCS2	288 (251, 330)	315 (270, 395)	.050	57 [43–70]	62 (47–77)	57 (41–72)

Abbreviations: AUC, area under the curve; CAP, community-acquired pneumonia; LOOCV, leave-one-out cross-validation; TB, tuberculosis.

^a^Wilcoxon rank-sum test.

^b^Median (interquartile range).

### The Performance of Protein Combinations for Diagnostic Testing of Active TB

The combinations of proteins 2 to 4 proteins were evaluated before and after the LOOCV (Supplementary Table 2). The proteins SULT4A1, WASF3, and CYTOb561, differentially expressed in TB versus CAP patients, yielded good diagnostic testing performances when combined after the LOOCV (Table 3). Two proteins-based combinations, SULT4A1-WASF3 and SULT4A1-CYTOb561, increased the performance of diagnostic testing of active TB from 70% to up to 79% (Table 3) compared to the performance of the single proteins SULT4A1 and WASF3. Three and 4 proteins-based combinations of SULT4A1-WASF3-CYTOb561 and SULT4A1-WASF3-KLCR4-CYTOb561 performed at 81% and 82%, respectively (Table 3).

**Table 3.** Performance of Diagnostic Testing of Combinations of Proteins

Model	Combinations	AUC After LOOCV
Model with 2 proteins	SULT4A1, CYTOb561	79 [68–89]
	SULT4A1, WASF3	78 [66–88]
	KLCR4, SPTLC1	75 [64–86]
	SULT4A1, FAM107B	75 [63–86]
	SULT4A1, SPTLC1	73 [60–84]
	SULT4A1, KLCR4	72 [59–83]
	WASF3, CYTOb561	71 [59–83]
	SPTLC1, CYSTEIN	71 [59–82]
	KLCR4, FAM107B	71 [58–82]
	SULT4A1, BIN1	70 [57–82]
Model with 3 proteins	SULT4A1, WASF3, CYTOb561	81 [73–88]
	SULT4A1, KLCR4, CYTOb561	79 [72–86]
	KLCR4, SPTLC1, CYTOb561	79 [71–86]
	SULT4A1, CYTOb561, BIN1	77 [70–84]
	SULT4A1, WASF3, FAM107B	77 [68–85]
	SULT4A1, FAM107B, CYTOb561	76 [69–83]
	SULT4A1, WASF3, KLCR4	76 [68–84]
	SULT4A1, SPTLC1, CYTOb561	76 [68–84]
	SULT4A1, CYTOb561, SORCS2	76 [68–84]
	KLCR4, SPTLC1, CYSTEIN	76 [68–84]
Model with 4 proteins	SULT4A1, WASF3, KLCR4, CYTOb561	82 [76–88]
	SULT4A1, KLCR4, CYTOb561, BIN1	82 [76–87]
	SULT4A1, KLCR4, CYTOb561, SORCS2	82 [76–87]
	KLCR4, SPTLC1, CYSTEIN, CYTOb561	81 [76–87]
	WASF3, KLCR4, CYTOb561, SORCS2	81 [75–86]
	SULT4A1, WASF3, CYTOb561, BIN1	81 [74–86]
	KLCR4, SPTLC1, CYSTEIN, SORCS2	80 [74–86]
	KLCR4, SPTLC1, CYTOb561, SORCS2	80 [74–86]
	SULT4A1, KLCR4, FAM107B, CYTOb561	80 [73–85]
	SULT4A1, KLCR4, CYSTEIN, CYTOb561	80 [73–85]

AUC was computed using the random forest model and LOOCV. The AUC after LOOCV is presented in Table 2.

Abbreviations: AUC, area under the curve; LOOCV, leave-one-out cross-validation.

### Selected Candidates' Biomarkers as Diagnostic Testing Tools for Active TB

Of 13 proteins reported in the current manuscript, 2 single proteins met our core selection criteria: (1) their concentration measured in serum was differentially expressed in TB compared to CAP patients; and (2) these proteins showed performance at least comparable to smear microscopy in distinguishing TB diseases from pneumonia of other causes; area under the curve (AUC) ≥ 70% with a lower limit of confidence interval >50%. SULT4A1: AUC (95% CI) = 70% (57–82) and WASPF3: AUC (95%CI) = 71% (60–82) met the selection criteria as hepcidin and creatine kinase band B, which were previously reported [12]. For the combination of the 3 proteins SULT4A1, WASF3, CYTOb561, AUC (95% CI) = 81% (73–88) met the selection criteria, including differential expression in TB versus CAP patients and an AUC > 80% with a lower limit of CI > 70% and upper limit >83%.

## DISCUSSION

In our study, sulfotransferase 4A1 (SULT4A1), Wiskott-Aldridge syndrome protein family member 3 (WASPF3), family with sequence similarity 107B (FAM107B), serine palmitoyl transferase, long chain base subunit 1 (SPLTC1), cytochrome b 561 (CYTO b 561), and sortilin-related VPS10 domain-containing receptor 2 (SORCS2) were differentially expressed in TB compared to CAP patients. Using the inferential random forest model combined with the LOOCV method, we selected SULT4A1 and WASPF3 single proteins as candidate biomarkers for the diagnosis of active TB cases. Both performed at least equally to the smear microscopy and, therefore, may be used as PoC diagnostic testing tools to screen TB-suspected patients in resource-constrained and remote areas. The SULT4A1, WASPF3, and CYTOb561 combination performed better than the smear microscopy. It encompassed the performance rate of rapid diagnostic tests recommended by the WHO, supporting its selection as a candidate for complementary rapid diagnostic testing tools for active TB cases.

The SULT4A1 belongs to the superfamily of sulfotransferases cytosolic enzymes that sulfonate endogenous and exogenous substrates such as hormones, neurotransmitters, and small metabolites, as well as exogenous xenobiotics by transferring the sulfonate group from 3′-phosphoadenosine 5′-phosphosulfate to different endogenous and exogenous substrates [14]. SULT4A1 distribution and functions are primarily located in the brain. However, it has been found in other organs, such as the kidney, lung, liver, and heart [15]. Although there is little or no data about the involvement of SULT4A1 in TB disease, evidence suggests that the cellular sulfonation pathway controls the main steps of intracellular viral replication and that cytosolic SULT1A1a modulates explicitly the replication of the HIV-1 virus by replication reverse transcription in primary human monocyte-derived macrophages [16]. A similar implication of SULT4A1 in the sulfonation of xenobiotics may involve human monocyte-derived macrophages to protect the human cells against oxidative stress during TB disease [17] or to restrict the growth of Mtb in human macrophages [18]. Remarkably, 2 mycobacterial sulfotransferases, stf1 and stf10, highly similar to mammalian sulfotransferases, have been identified for *M tuberculosis*, *M leprae, M smegmatis,* and *M avium.* Stf1 and stf10 add to the list of mycobacterial eukaryotic-like protein families and may also interact with the human host immunity to TB [19].

The WASPF3 is a cytosolic protein of 502 amino acids. WASP functions depend mainly on regulating the actin cytoskeleton in hematopoietic cells and encompass myeloid and lymphoid cell migration, receptor signalling, phagocytosis, and cytotoxicity. Actine cytoskeleton regulation–independent functions of WASPF3 imply nuclear transcription and comprise T-cell differentiation, memory B-cell activation through transcription of B-cell co-receptor CD19, transcription of inflammatory cytokines, and transcriptional regulation of myeloid cells [20]. It has been consistently reported that intracellular pathogens, including *Mycobacterium* spp, *Listeria monocytogenes*, *Shigella flexneri*, and *Rickettsia rickettsii*, subvert the host cytoskeleton by using actin-based motility to spread across host cells to establish and maintain the infection [21].

One study has demonstrated actin tail formation by *Mycobacterium marinum* in the cytoplasm of macrophages that involve WASP and neuronal WASP, suggesting that both proteins may be leading the motility of mycobacteria in macrophages and their intercellular spread by favoring actin-based motility [22].

As hepcidin, which reflects human macrophage-Mtb pathogen interactions through the regulation of iron metabolism and inflammation and for which we have reported a high performance to distinguish TB disease from pneumonia of other causes, the differential expression of SULT4A1 and WASPF via the sulfonation of Mtb products, the regulation of the actin cytoskeleton in the macrophages, may also be due to human macrophages-Mtb interactions contributing to host defense. However, the expression of SULT4A1 and WASPF3 may also reflect the mechanisms of *Mtb* evasion from the host immune system through the elaboration of mycobacterial eukaryotic-like proteins mimicking the human SULT4A1 or the use of the WASPF3 to build actin tails, which facilitate the mobility and spread of mycobacteria in the cytoplasm of macrophages. In addition, the selected host biomarker candidates may be involved in host pathogens' interactions with many other infectious agents: hepcidin, for instance, is known to play a role in malaria immunity. The dual pattern of host biomarkers involved in Mtb disease, including the defense of the host or the subversion of the host immune system by the pathogen and their potential roles in other infectious diseases necessitate a fine-tuned understanding of the kinetics of their expression in TB patients, in patients experiencing infections like malaria, and in healthy controls.

Combining the 2 single proteins, SULT4A1-WASPF3, which met our selection criteria substantially, augments the performance in discriminating between TB and CAP patients, from 70% as single proteins to 78%. The combination SULT4A1-CYTOb561 has a slightly better performance than SULT4A1-WASPF3, reaching more than 79% performance. CYTOb561 does not meet the selection criteria as a single protein; its performance was below 70% despite being differentially expressed by TB and CAP patients. Cytochromes b561 (Cyts b561) are a family of intrinsic membrane proteins. Cyts b561 are involved in the transmembrane transport of electrons under the mediation of ascorbate. A Cyt b561 named LCytb (for lysosomal Cytb561) is expressed in the endosomal-lysosomal membrane of the lung, spleen, thymus, testes, and placenta tissues. In the lung, the protein is described in the alveolar macrophages. A study showed decreased expression of Lcytb in macrophages exposed to pathogenic *Escherichia coli* strains, suggesting its role in regulating iron availability for intracellular bacteria [23]. However, all combinations of the 2 proteins tested had a lower CI limit below 70% and/or crossed the ones of single proteins. The combinations of SULT4A1, WASPF3, and CYTOb561 were the best-performing combination of only differentially expressed proteins in TB versus CAP patients. Theoretically, the combination reflects the intracellular motility of Mtb (WASPF3), the interactions between the human macrophages and Mtb pathogen leading to the protection of lung cells against oxidative stress (SULT4A1) and intracellular regulation of iron (CYTOb561).

## CONCLUSION

The candidate biomarkers from our selection process include SULT4A1 and WASPF3 as single proteins and SULT4A1, WASPF3, and CYTOb561 in combination. However, several unanswered questions and limitations of a small sample size and a cross-sectional study design must be addressed. Further steps will address (1) the value of each selected candidate as a screening and triage point-of-care tool in community and primary health centers to inform the need for further referral for sputum-based molecular and mycobacterial culture testing; (2) the assessment of the value of selected candidate biomarkers in the algorithm, including gold standard TB diagnostic tests, to improve access to TB diagnosis of people in resource-limited settings; (3) the assessment of candidate biomarkers to monitor the effectiveness of TB treatments; and (4) the kinetics of selected candidate biomarkers in TB patients versus other infectious diseases patients and healthy individuals.

## Supplementary Data

Supplementary materials are available at *Open Forum Infectious Diseases* online. Consisting of data provided by the authors to benefit the reader, the posted materials are not copyedited and are the sole responsibility of the authors, so questions or comments should be addressed to the corresponding author.

## Supplementary Material

ofae399_Supplementary_Data

## Notes

***Author's contributions*.** P. N. E., B. R. A., and A. S. T. designed the study. B. R. A. managed participants. P. N. E. and E. M. performed the experiments. F. L. M., P. N. E., and A. S. T. analyzed the data. P. N. E., A. S. T., and F. L. M. wrote the first draft. F. L. M., P. N. E., A. S. T., and M. P. G. contributed to data interpretation and the writing of the final version of the manuscript. All authors reviewed the manuscript's final submission for publication.

***Patient consent statement*.** The Institutional Ethics Committee of CERMEL approved the study. All study participants gave written informed consent to participate in the study.

***Financial support*.** This work was supported by the European and Developing Countries Clinical Trials Partnership (EDCTP-TMA-SF-1946-VARSAF). Marting P. Grobusch's research group provided cofunding.
