# Supplementary material for: Single and Combined Serum Proteins Expressed in TB Infection are Candidates for Point-of-care Diagnostic Testing of Active TB Patients in Lambaréné, Gabon
Source: Open Forum Infect Dis. 2024 Jul 13;11(8):ofae399. doi: 10.1093/ofid/ofae399 (PMC11303003; doi:10.1093/ofid/ofae399)
Supplement: ofae399_Supplementary_Data [file ofae399_supplementary_data.docx]

**Supplement to the manuscript**

Paulin N. Essone^1,3^*, Fabrice Lotola-Mougeni^1^, Bayode R. Adegbite^1,2,4^, Kossiwa Kokou^1^ , Otogo N'Nang E^1^, Eddy Mabicka^1^, Ayodele Alabi^1^, Joel F.D. Siawaya^3^, Peter G. Kremsner^1,2^, Martin P. Grobusch^1,2,4^, Selidji T. Agnandji^1,2,5,^*

**Single and combined serum proteins expressed in TB infection are candidates for point-of-care diagnostic testing of active TB patients in Lambaréné, Gabon**

Short title:

**Serum** **candidates' biomarkers for diagnostic testing of active TB cases**

**Materials and Methods**

***ELISA to determine the concentration of proteins in serum of TB and CAP patients***

Serum levels of the following biomarkers were selected for ELISA: SULT4A1, WASF3, KLCR4, TRGC1, FAM107B, SPTLC1, CYSTEIN, CYTOb561, BIN1, NRG1, SORCS2, ASAH2, C11ORF83, RNASE7, HOGA1, OLFM4 and MMRN2. The levels of these biomarkers were determined with commercial ELISA kits (MyBioSource, San Diego, USA). ELISAs were performed according to the manufacturer's instructions for the individual kits. Briefly, standards, blanks, samples, and 100 μL of HRP-conjugated reagents were added to the appropriate pre-coated well plates and incubated at 37^0^C for one hour. The plates were washed four times before adding 50 μL of chromogen solution A and 50 μL of chromogen solution B. The plates were incubated at 37^0^C for 15 minutes. The reactions were stopped with 50 μL of stopping solution before reading at 45nm using an ELISA reader.

***Statistical analysis***

The median with IQR and the percentage were presented to describe each continuous or categorical variable's distribution. The concentration of each protein was compared between TB and CAP patients using the Manh-Whitney test. For the classification or diagnosis of TB and CAP patients, the random Forest model was used, and the AUC was computed from the model to evaluate its performance: the ability of the model, by considering different cut-offs, to distinguish TB and CAP patients correctly on average. The model was validated using leave-one-out cross-validation (LOOCV) as the sample size was small. The LOOCV iteratively trained the data on the full data except for one observation, which will be used as test data for the trained model. Data underwent log transformation. Data were analysed using the R software, version 4.0.3

**Results**

**Table S1.** Concentration and sensitivity, specificity, AUC evaluation of active TB diagnostic testing of 13 proteins before and after LOOCV.

|  | | | | Before LOOCV | | | After LOOCV | | |
| --- | --- | --- | --- | --- | --- | --- | --- | --- | --- |
| Markers | TB patients | CAP patients | p-value^2^ | AUC | Sensitivity 95% CI | Specificity 95% CI | AUC [95% CI] | Sensitivity 95% CI | Specificity 95% CI |
| ^3^SULT4A1 | 9 (6, 33) | 5 (4, 9) | <0.001 | 98 | 99 (95 - 100) | 96 (90 - 100) | 70[ 57 - 82 ] | 70 (55 - 84) | 57 (41 - 72) |
| C11orf83 | 0.00 (0.00, 0.00) | 0.00 (0.00, 0.00) | 0.4 | 60 | 14 (4 - 26) | 99 (95 - 100) | 12 | 12 (3 - 22) | 96 (90 - 100) |
| MMRN2 | 0.0000 (0.0000, 0.0000) | 0.0000 (0.0000, 0.0000) | 0.6 | 54 | 99 (95 - 100) | 7 (0 - 14) | 0 | 36 (21 - 51) | 1 (0 - 5) |
| ^3^WASF3 | 470 (365, 1,000) | 350 (284, 454) | 0.003 | 96 | 99 (95 - 100) | 93 (86 - 100) | 71[ 60 - 82 ] | 67 (52 - 81) | 64 (49 - 79) |
| ^3^KLCR4 | 56 (36, 93) | 78 (20, 192) | 0.7 | 97 | 93 (86 - 100) | 99 (95 - 100) | 59[ 46 - 72 ] | 54 (38 - 69) | 59 (44 - 74) |
| TRGC1 | 0 (0, 6,485) | 0 (0, 7,568) | 0.8 | 82 | 43 (28 - 59) | 99 (95 - 100) | 27 | 20 (8 - 32) | 80 (68 - 92) |
| FAM107B | 239 (160, 575) | 141 (126, 234) | 0.002 | 100 | 96 (90 - 100) | 93 (86 - 100) | 63[ 50 - 76 ] | 49 (33 - 64) | 62 (47 - 77) |
| SPTLC1 | 8 (4, 24) | 2 (2, 8) | 0.001 | 97 | 93 (86 - 100) | 99 (95 - 100) | 64[ 51 - 76 ] | 57 (41 - 72) | 57 (41 - 72) |
| CYSTEIN | 48 (44, 55) | 47 (44, 51) | 0.3 | 97 | 91 (82 - 99) | 96 (90 - 100) | 51[ 38 - 64 ] | 57 (41 - 72) | 57 (41 - 72) |
| CYTOb561 | 27 (20, 44) | 21 (0, 38) | 0.049 | 89 | 83 (71 - 94) | 99 (95 - 100) | 59[ 46 - 72 ] | 49 (33 - 64) | 64 (49 - 79) |
| BIN1 | 99 (49, 191) | 94 (47, 195) | 0.8 | 100 | 99 (95 - 100) | 91 (82 - 99) | 49[ 35 - 62 ] | 49 (33 - 64) | 49 (33 - 64) |
| NRG1 | 3.95 (3.06, 4.78) | 3.16 (1.40, 4.30) | 0.057 | 98 | 93 (86 - 100) | 99 (95 - 100) | 55[ 42 - 68 ] | 54 (38 - 69) | 57 (41 - 72) |
| SORCS2 | 288 (251, 330) | 315 (270, 395) | 0.050 | 100 | 99 (95 - 100) | 99 (95 - 100) | 57[ 43 - 70 ] | 62 (47 - 77) | 57 (41 - 72) |
| ^1^Median (IQR) | | | | | | | | | |
| ^2^Wilcoxon rank sum test | | | | | | | | | |

Median levels of serum analytes and their accuracy in diagnosing TB disease as a single host marker. P-values were calculated using the Mann-Whitney U test. AUC = area under the receiver operator characteristics curve. 95% CI = 95% confidence interval. Biomarkers with P value ≤0,05 and Area Under the Curve (AUC) ≥ 70% are in bold.

^3^ All proteins are expressed in ng/ml except for SULT4A1, WASF3, KLCR4, which are expressed in pg/ml

| **Table S2.** Accuracy and AUC of active TB diagnostic testing of combinations of two to four proteins before and after LOOCV. | | **Before LOOCV** | | | | **After LOOCV** | | | |
| --- | --- | --- | --- | --- | --- | --- | --- | --- | --- |
| **Model** | **Combinations** | **TBcase (%)** | **NoTBcase (%)** | **Accuracy [95 % CI]** | **AUC** | **TBcase (%)** | **NoTBcase (%)** | **Accuracy (%)** | **AUC [95% CI]** |
| Model with 2 analytes | SULT4A1, CYTOb561 | 100.0 | 100.0 | 100 [95.1-100] | 100 | 70.3 | 75.7 | 73 [61.4-82.6] | 79 [ 68 - 89 ] |
|  | SULT4A1, WASF3 | 94.6 | 100.0 | 97.3 [90.6-99.7] | 98 | 70.3 | 78.4 | 74.3 [62.8-83.8] | 78 [ 66 - 88 ] |
|  | KLCR4, SPTLC1 | 100.0 | 100.0 | 100 [95.1-100] | 100 | 70.3 | 70.3 | 70.3 [58.5-80.3] | 75 [ 64 - 86 ] |
|  | SULT4A1, FAM107B | 97.3 | 100.0 | 98.6 [92.7-100] | 100 | 70.3 | 73.0 | 71.6 [59.9-81.5] | 75 [ 63 - 86 ] |
|  | SULT4A1, SPTLC1 | 97.3 | 100.0 | 98.6 [92.7-100] | 100 | 70.3 | 67.6 | 68.9 [57.1-79.2] | 73 [ 60 - 84 ] |
|  | SULT4A1, KLCR4 | 100.0 | 100.0 | 100 [95.1-100] | 100 | 70.3 | 64.9 | 67.6 [55.7-78] | 72 [ 59 - 83 ] |
|  | WASF3, CYTOb561 | 94.6 | 94.6 | 94.6 [86.7-98.5] | 100 | 62.2 | 67.6 | 64.9 [52.9-75.6] | 71 [ 59 - 83 ] |
|  | SPTLC1, CYSTEIN | 100.0 | 100.0 | 100 [95.1-100] | 100 | 54.1 | 70.3 | 62.2 [50.1-73.2] | 71 [ 59 - 82 ] |
|  | KLCR4, FAM107B | 100.0 | 97.3 | 98.6 [92.7-100] | 100 | 62.2 | 70.3 | 66.2 [54.3-76.8] | 71 [ 58 - 82 ] |
|  | SULT4A1, BIN1 | 100.0 | 100.0 | 100 [95.1-100] | 100 | 70.3 | 75.7 | 73 [61.4-82.6] | 70 [ 57 - 82 ] |
| Model with 3 analytes | SULT4A1, WASF3, CYTOb561 | 97.3 | 100.0 | 98.6 [92.7-100] | 100 | 74.3 | 73.0 | 73.6 [65.8-80.5] | 81 [ 73 - 88 ] |
|  | SULT4A1, KLCR4, CYTOb561 | 100.0 | 100.0 | 100 [95.1-100] | 100 | 71.6 | 71.6 | 71.6 [63.6-78.7] | 79 [ 72 - 86 ] |
|  | KLCR4, SPTLC1, CYTOb561 | 100.0 | 100.0 | 100 [95.1-100] | 100 | 70.3 | 68.9 | 69.6 [61.5-76.9] | 79 [ 71 - 86 ] |
|  | SULT4A1, CYTOb561, BIN1 | 100.0 | 100.0 | 100 [95.1-100] | 100 | 63.5 | 73.0 | 68.2 [60.1-75.6] | 77 [ 70 - 84 ] |
|  | SULT4A1, WASF3, FAM107B | 97.3 | 100.0 | 98.6 [92.7-100] | 100 | 75.7 | 73.0 | 74.3 [66.5-81.1] | 77 [ 68 - 85 ] |
|  | SULT4A1, FAM107B, CYTOb561 | 100.0 | 100.0 | 100 [95.1-100] | 100 | 67.6 | 70.3 | 68.9 [60.8-76.3] | 76 [ 69 - 83 ] |
|  | SULT4A1, WASF3, KLCR4 | 97.3 | 100.0 | 98.6 [92.7-100] | 100 | 71.6 | 67.6 | 69.6 [61.5-76.9] | 76 [ 68 - 84 ] |
|  | SULT4A1, SPTLC1, CYTOb561 | 100.0 | 100.0 | 100 [95.1-100] | 100 | 67.6 | 64.9 | 66.2 [58-73.8] | 76 [ 68 - 84 ] |
|  | SULT4A1, CYTOb561, SORCS2 | 100.0 | 100.0 | 100 [95.1-100] | 100 | 67.6 | 73.0 | 70.3 [62.2-77.5] | 76 [ 68 - 84 ] |
|  | KLCR4, SPTLC1, CYSTEIN | 100.0 | 100.0 | 100 [95.1-100] | 100 | 67.6 | 74.3 | 70.9 [62.9-78.1] | 76 [ 68 - 84 ] |
| Model with 4 analytes | SULT4A1, WASF3, KLCR4, CYTOb561 | 100.0 | 100.0 | 100 [95.1-100] | 100 | 76.6 | 76.6 | 76.6 [70.4-82] | 82 [ 76 - 88 ] |
|  | SULT4A1, KLCR4, CYTOb561, BIN1 | 100.0 | 100.0 | 100 [95.1-100] | 100 | 78.4 | 75.7 | 77 [70.9-82.4] | 82 [ 76 - 87 ] |
|  | SULT4A1, KLCR4, CYTOb561, SORCS2 | 100.0 | 100.0 | 100 [95.1-100] | 100 | 68.5 | 73.0 | 70.7 [64.3-76.6] | 82 [ 76 - 87 ] |
|  | KLCR4, SPTLC1, CYSTEIN, CYTOb561 | 100.0 | 100.0 | 100 [95.1-100] | 100 | 68.5 | 81.1 | 74.8 [68.5-80.3] | 81 [ 76 - 87 ] |
|  | WASF3, KLCR4, CYTOb561, SORCS2 | 100.0 | 100.0 | 100 [95.1-100] | 100 | 75.7 | 70.3 | 73 [66.6-78.7] | 81 [ 75 - 86 ] |
|  | SULT4A1, WASF3, CYTOb561, BIN1 | 100.0 | 100.0 | 100 [95.1-100] | 100 | 71.2 | 74.8 | 73 [66.6-78.7] | 81 [ 74 - 86 ] |
|  | KLCR4, SPTLC1, CYSTEIN, SORCS2 | 100.0 | 100.0 | 100 [95.1-100] | 100 | 74.8 | 68.5 | 71.6 [65.2-77.5] | 80 [ 74 - 86 ] |
|  | KLCR4, SPTLC1, CYTOb561, SORCS2 | 100.0 | 100.0 | 100 [95.1-100] | 100 | 75.7 | 76.6 | 76.1 [70-81.6] | 80 [ 74 - 86 ] |
|  | SULT4A1, KLCR4, FAM107B, CYTOb561 | 100.0 | 100.0 | 100 [95.1-100] | 100 | 73.9 | 67.6 | 70.7 [64.3-76.6] | 80 [ 73 - 85 ] |
|  | SULT4A1, KLCR4, CYSTEIN, CYTOb561 | 100.0 | 100.0 | 100 [95.1-100] | 100 | 73.0 | 73.9 | 73.4 [67.1-79.1] | 80 [ 73 - 85 ] |
